# Recent Trends in the Clinicopathological Features of Thyroid Nodules in Pediatric Patients: A Single Tertiary Center Experience over 25 Years

**DOI:** 10.1155/2019/1829043

**Published:** 2019-12-20

**Authors:** Cheong-Sil Rah, Won Woong Kim, Yu-mi Lee, Won Gu Kim, Dong Eun Song, Ki-Wook Chung, Seong Chul Kim, Suck Joon Hong, Tae-Yon Sung

**Affiliations:** ^1^Department of Surgery, Asan Medical Center, University of Ulsan College of Medicine, 88 Olympic-ro 43-gil, Songpa-gu, Seoul 05505, Republic of Korea; ^2^Department of Internal Medicine, Asan Medical Center, University of Ulsan College of Medicine, 88 Olympic-ro 43-gil, Songpa-gu, Seoul 05505, Republic of Korea; ^3^Department of Pathology, Asan Medical Center, University of Ulsan College of Medicine, 88 Olympic-ro 43-gil, Songpa-gu, Seoul 05505, Republic of Korea; ^4^Department of Pediatric Surgery, Asan Medical Center, University of Ulsan College of Medicine, 88 Olympic-ro 43-gil, Songpa-gu, Seoul 05505, Republic of Korea

## Abstract

**Background:**

The trends in pediatric patients having thyroid nodules have not been well evaluated. Here, we analyzed the clinicopathological features of the children who have presented with thyroid nodules at our center over several decades in order to determine a change of trend.

**Materials and Methods:**

We analyzed 215 pediatric patients who had undergone a thyroidectomy between 1990 and 2014 at our single tertiary center. The clinicopathological features were analyzed according to age, sex, and the year of diagnosis.

**Results:**

The most common reason for hospital admission was a palpable anterior neck mass (76.7%). The males in the patient cohort were younger than the females (13 vs. 15 years old, *p* < 0.05). The female patients increased from 50.0% to 83.0% with age (*p* < 0.05). The rate of malignancy did not increase with age (*p* < 0.05). However, the malignancy rate was higher in the more recently seen patients (23.8% during the early study period and 86.8% in the late study period; *p* < 0.05).

**Conclusion:**

Following a thyroidectomy in children with thyroid nodules, there was no change in the rate of detection of thyroid cancer over time with age, although the detected frequency of malignancy has increased in more recent patients. Therefore, early thyroid nodule detection for malignant screening is likely to be required for pediatric patients in the near future.

## 1. Introduction

Thyroid nodules are frequently detected in young people, and thyroid cancer is common in younger women showing an increasing incidence over the last few decades [1–3]. Among the various known kinds of pediatric endocrine malignancy, well-differentiated thyroid cancer (WDTC) is the most common [4–6]. The clinical behaviors of WDTC in children differ from those in adults as the pediatric patients tend to have more cervical lymph node (LN) metastases, extrathyroidal extensions, and lung metastases [6–10].

The early detection of thyroid nodules in adult patients has contributed to the increased diagnosis of thyroid cancer and has improved the clinical outcomes in these patients [9, 11–13]. In pediatric thyroid nodule patients, the American Thyroid Association recommends ultrasonography (US) and fine needle aspiration (FNA) for the evaluation of thyroid nodules [14–16]. More accurate diagnoses of the nodules have, therefore, been made possible, although the number of unnecessary examinations of benign nodules has also increased [17–19].

In our current study, we focused on observing the changes in the clinicopathological features of pediatric patients with thyroid nodules who were admitted to our hospital for a thyroidectomy between 1990 and 2014. We retrospectively evaluated the trends in this patient cohort in accordance with their age, sex, and year of diagnosis.

## 2. Materials and Methods

### 2.1. Study Population

This retrospective cohort study included 215 pediatric patients who were admitted to Asan Medical Center, Seoul, Korea, between January 1990 and December 2014 for a thyroidectomy due to suspected thyroid nodules. This study was approved by the Institutional Review Board of Asan Medical Center (no. 2015-0844). Data were obtained from the prospectively maintained endocrine surgery database available at Asan Medical Center, and the requirement for informed consent from each patient was waived due to the noninterventional nature of the study. The patients were referred to our hospital either by local doctors or by the patient's parent need. Pediatric patients were defined as age ≤18 years. The study patients were classified by age as follows: 0 to 10 years (*n* = 24), 11 to 15 years (*n* = 79), and 16 to 18 years (*n* = 112). They were also classified according to the period of diagnosis: 1990–1995 (*n* = 42), 1996–2000 (*n* = 50), 2001–2005 (*n* = 40), 2006–2010 (*n* = 38), and 2011–2014 (*n* = 45). The endocrinologists, radiologists, pathologists, and surgeons involved in the management of the study patients over the 25 year period had remained mainly the same. The same management protocol at our institution for these patients is applied across the different departments in order to achieve a consistency in clinical examination and patient care. In this study, all of the patients had no familial or syndromic thyroid cancer presentation. Pathologic review was performed on all of the specimens.

### 2.2. Management Protocol and Outcome Measurements

All of the study patients presenting at our hospital with suspected thyroid nodules were evaluated by physical examination, thyroid function test (T3, free T4 and thyroid-stimulating hormone), neck US, and FNA when in need. The initial cytology results after FNA were categorized in accordance with the Bethesda Thyroid Cytopathology Reporting System and were reviewed by pathologists with consistency [20]. Clinicopathological features such as age, sex, year of diagnosis, chief complaint related to the thyroid nodules necessitating a hospital visit, initial FNA cytology, final pathology, and overall follow-up duration were evaluated.

### 2.3. Statistical Analysis

All statistical analyses were conducted using R (version 3.1.0) and the R library package (R Foundation for Statistical Computing, Vienna, Austria; http://www.R-project.org). The continuous variables were presented as a mean with standard deviation (SD) or medians with the interquartile range (IQR). Categorical variables were presented as numbers with percentages. The student *t*-test and Wilcoxon rank-sum test were used to compare the continuous variables, and the *x*^2^ test and Fisher's exact test were used to compare the categorical variables. *p* values <0.05 were considered to indicate statistical significance.

## 3. Results

### 3.1. Baseline Features of the Pediatric Study Patients with Thyroid Nodules

The baseline clinical and pathological features of the 215 children in our current series who had thyroid nodules and who underwent a thyroidectomy are listed in Table 1. The mean age of this whole study cohort was 15 years. In the age subgroups, 3 patients (1.4%) were less than 5 years of age, 21 patients (9.8%) were between 6 and 10 years, 79 patients (36.7%) were between 11 and 15 years, and 112 patients (52.1%) were between 16 and 18 years of age. There were 166 females (77.2%) in the total patient cohort. The median overall follow-up duration was 75 months.

**Figure 1:**
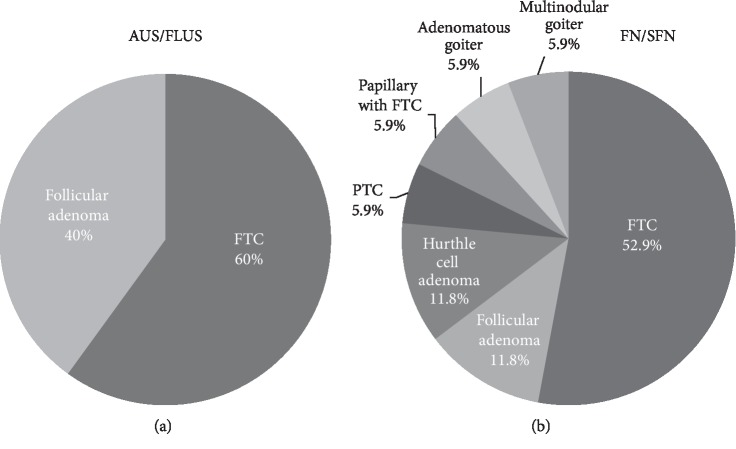
Distribution of the final pathology according to the initial cytology results of atypia of undetermined significance or follicular lesions of undetermined significance (AUS/FLUS) and follicular neoplasm or suspicion of follicular neoplasm (FN/SFN). (a) 15 patients with AUS/FLUS. (b) 17 patients with FN/SFN. (PTC, papillary thyroid cancer; FTC, follicular thyroid cancer).

The most common causes of hospital admission among the study patients were a palpable anterior neck mass (*n* = 165, 76.7%), followed by an incidentaloma (*n* = 21, 9.8%). There were no recorded reasons for the hospital presentation in 14 patients (6.5%). Fifty-four patients (25.1%) underwent thyroidectomy without a preoperative cytology evaluation. Among them, one patient presented with malignancy in the final pathology. The malignant patient was diagnosed with papillary thyroid cancer (PTC). Among non-FNA patients, diffuse hyperplasia or diffuse hyperplasia/graves at the final pathology accounted for the largest percentage of each period. By initial cytology, 23 out of the 161 FNA patients (14.3%) presented with benign cytology results, and 92 (57.1%) presented with a malignancy. Cytology results displaying atypia of undetermined significance or follicular lesions of undetermined significance (AUS/FLUS) presented in 15 of these patients (9.3%), and a follicular neoplasm (FN) or a suspicion of follicular neoplasm (SFN) was found in 17 patients (10.6%). Among 15 patients of AUS/FLUS, 9 (60%) presented with follicular thyroid cancer (FTC), and 6 (40%) presented with follicular adenoma. Among 17 FN/SFN patients, 9 (52.9%) were FTC. There was 1 patient (5.9%) with PTC and papillary with FTC each. The remaining 6 were benign, including 2 (11.8%) goiter patients, 2 (11.8%) follicular adenoma patients, and 2 (11.8%) with Hurthle cell adenoma (Figure 1).

In the final pathology assessments after the thyroidectomy, 87 patients (40.5%) were diagnosed as benign and 128 patients (59.5%) with malignant disease. In the benign disease patients, Graves' disease including diffuse hyperplasia was evident in 37 patients (42.5%) and adenomatous goiter or nodular hyperplasia in 26 patients (29.9%). In patients with malignant diseases, PTC was present in 106 (82.8%) patients. The overall median tumor size according to pathology was 2.5 cm.

**Figure 2:**
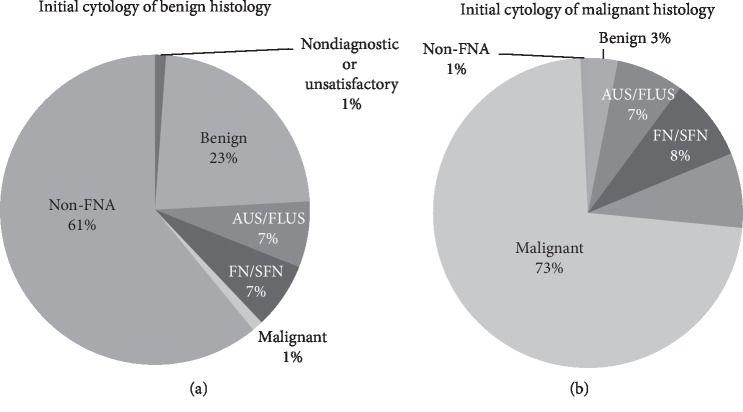
Distribution of the initial cytology according to the final pathology results as benign or malignant. Initial cytology of (a) benign histology and (b) malignant histology (FNA, fine needle aspiration; AUS/FLUS, atypia of undetermined significance or follicular lesions of undetermined significance; FN/SFN, follicular neoplasm or suspicion of follicular neoplasm).

To add information regarding the proportion of the initial cytology distributions of the final pathology, we assessed the FNA results of the final benign and malignant histology patients. For the initial cytology of the final benign histology, 1 patient presented as malignant in the initial cytology. For the initial cytology of the final malignant histology, 93 patients (73%) presented with malignancy and only 4 patients (3%) had benign (Figure 2).

### 3.2. Clinicopathological Features according to Patient Age

The patients were divided into three age groups as indicated in Table 2: age group 1, 0 to 10 years (*n* = 24); group 2, 11 to 15 years (*n* = 79); and group 3, 16 to 18 years (*n* = 112). The proportion of females dramatically increased with age as follows: 50.0% in group 1, 77.2% in group 2, and 83.0% in group 3 (*p*=0.002). There was no difference between these three groups in terms of the period of diagnosis, the chief complaint necessitating their hospital visit, final pathology after thyroidectomy, or the tumor size as determined by pathology (*p* > 0.05). A palpable neck mass was the most common cause of their hospital admission (>75.0%) in all of the patient groups.

The initial cytology showed differences between the groups (*p* < 0.05). In these results, the benign rate was higher in group 1 (25.0%) compared with groups 2 (11.9%) or 3 (13.5%). The rate of malignancy was higher in all three groups with a clear increase with age (group 1 = 30.0%, group 2 = 56.7%, and group 3 = 64.9%). The final pathology results after a thyroidectomy indicated comparable benign and malignant rates in age group 1 (50% vs 50%), but higher rates of malignancy in groups 2 and 3 (65.8% and 57.1%, respectively), although this did not differ significantly. In the benign pathology patients, adenomatous goiter or nodular hyperplasia was the most frequently diagnosed disorders in age groups 1 (50.0%) and 2 (40.7%), with Graves' disease (diffuse hyperplasia) being the most common in group 3 (62.5%). In the patients with malignant pathology, PTC was the most common cancer present throughout the groups. However, in group I, FTC showed a higher rate (25.0%) than that in the other groups (group 2 = 15.4% and group 3 = 15.6%).

### 3.3. Clinicopathological Features according to Sex

The patients were further classified according to their sex (Table 3). The mean age of the females was 15 years and of the males was 13 (*p*=0.001). A high proportion of the children in both groups presented with an anterior neck palpable mass as the chief complaint necessitating a hospital visit (females 77.7% and males 73.5%). FNA was more frequently performed in the males (85.7% vs. 71.7%). In the final pathology after thyroidectomy, Graves' disease (diffuse hyperplasia) was the most common benign disease (51.5%) in the females, whereas adenomatous goiter or nodular hyperplasia was the most common benign disease (38.1%) in the males. In the patients with malignant disease, PTC was most common in both sex, and there were no differences in terms of the tumor size as determined by pathology (*p* > 0.05).

### 3.4. Clinicopathological Features according to the Period of Diagnosis

The clinicopathological features of the 215 pediatric patients in our current series were stratified according to the period of diagnosis as follows: period I, 1990–1995; period II, 1996–2000; period III, 2001–2005; period IV, 2006–2010; and period V, 2011–2014 (Table 4). There were no statistically significant differences in age, sex, and the chief complaint leading to hospital admission between these period groups (*p* > 0.05) The most frequent cause of hospital admission was an anterior neck palpable mass, which presented in more than 60% of the children across the five diagnosis periods. However, this rate decreased in the later time periods, while the incidentaloma rates increased (period I, 88.1% vs. 0%; period II, 84.0% vs. 2.0%; period III, 82.5% vs. 7.5%; period IV, 68.4% vs. 15.8%; and period V, 60.0% vs. 24.4%, respectively).

There was also a significant difference in the FNA rate (*p* < 0.05) across these time periods. In period 1, only 40.5% of the children underwent FNA, but this rate showed a continual increase in the later time periods such that 91.1% of our study patients diagnosed in period V underwent this procedure. In terms of the initial cytology, 41.2% of the children diagnosed in period I presented as benign; however, this continuously decreased so that in period V, only 7.3% of the patients were found to be benign. In the final pathology, there were more benign patients in period 1 (76.2% vs. 23.8%), but this had dramatically reversed by period 4 in which malignant disease was predominant (13.2% vs. 86.8%, respectively).

## 4. Discussion

We have evaluated the trend changes over several decades in the clinicopathological features of pediatric patients with thyroid nodules who underwent thyroidectomy. A total of 215 children were enrolled in our current investigation who had been diagnosed, treated, and followed using a uniform protocol at our single tertiary institution. The most common causes of hospital presentation and requirement for thyroidectomy in this pediatric cohort were a palpable anterior neck mass followed by incidentaloma. Notably, the rate of incidentaloma has increased in the more recently diagnosed patients with a concomitant decrease seen in the palpable neck mass rate. The rate of malignant pathology presented no definite relationship with the age difference, but the rates were lower in patients less than 10 years of age and with a slight increase as the age cohort increased. However, the reported malignancy rate has clearly changed and appears to be far more common in the more recently diagnosed patients. These findings suggest that the rate of malignancy may be uniform in children with thyroid nodules over the past 25 years, but that there are significant increases in diagnosis in more recent years.

The incidence of detected thyroid nodules in young populations has been previously reported to show an increase in recent studies [3, 14, 21]. In our present study, a majority of the children visiting our hospital with suspected thyroid nodules requiring a thyroidectomy were between 16 and 18 years of age (52.1%), while only about 1 in 10 were younger than 10 years of age (11.2%). Interesting results were found regarding the male and female proportion. The proportion of male and female patients was 50.0%, respectively, between the ages 0 and 10. However, the female patients dramatically increased with the increase in age by 77.2% for group 2 and 83.0% for group 3 (*p*=0.002). These findings could relate to the general concept of thyroid nodules frequently being detected as a problematic condition in adult females compared with the adult males. In addition, the total pediatric population visiting our hospital each year shown a uniform pattern as time passes by and without noticeable increase.

The overall prevalence of palpable thyroid nodules in childhood is known to be lower than in adulthood, but the risk of malignancy is higher in pediatric patients (16% versus 5%) [22, 23]. In our current study, the rate of benign versus malignant lesions among the treated thyroid nodules in the pediatric study patients was nearly 50% in the early age group and had slightly increased in the older children. The malignancy rate did not differ according to sex (60.2% versus 57.1%), but the rate of benign versus malignant lesions in the final pathology assessment after thyroidectomy showed a large increase in the more recent periods of diagnosis, from 23.8% in 1990 to 86.8% in 2010. It is well known that PTC is most common in children [4, 24], and this rate was 82.8% in our current series. The clinical behavior of thyroid cancer in children is known to be more aggressive than that in adults [7, 9, 10]. In our previous reports, we also found that a young age was related to the aggressive pattern of thyroid cancer [25–27]. The early detection of thyroid cancer in pediatric patients might therefore also contribute to improved clinical outcomes, as seen in adult patients [12, 13]. American Thyroid Association pediatric guidelines have been recently announced and have recommended that the evaluation and treatment of pediatric thyroid nodules should be the same as in adults. They also recommended using the FNA approach before surgical treatment [14].

In our study, the malignancy rate of AUS/FLUS in FNA was 70%, and FN/SFN was nearly 65%. Most of the malignant history patients diagnosed with AUS/FLUS or FN/SFN were follicular variants of PTC. These results are higher than the malignant rate reported by the Bethesda system [28]. The reason could be that the patients who required FNA for the suspicion of malignant looking nodules under US of thyroid examination could have been referred to our institution, although it is difficult to distinguish between benign and malignant before the surgery.

A notable limitation of this study is the possibility of a selection bias due to the retrospective nature of the analyses. In addition, the data were obtained from our single tertiary center only, and our age groups were randomly assigned. However, we believe that we have provided a valuable assessment of recent trends in the clinicopathological features of children with thyroid nodules who have undergone thyroidectomy.

## 5. Conclusions

The number of children presenting with thyroid nodules requiring a thyroidectomy was uniform throughout the current study period. However, the number of female patients increased with age. The distribution of thyroid cancer detection was linear throughout the study age groups. The recorded proportion of malignancies has increased during the more recent diagnosis time periods. Since childhood thyroid cancer is more aggressive, early thyroid nodule detection for malignant screening is warranted in pediatric patients for a favorable prognosis.

## Figures and Tables

**Table 1:** Baseline features of the pediatric patients with thyroid nodules.

	Total (*n* = 215)	
Age, years (mean, SD)	15	±3.199
Age groups		
0–10	24	11.2%
11–15	79	36.7%
16–18	112	52.1%
Sex		
Females	166	77.2%
Males	49	22.8%
Period of diagnosis		
1990–1995	42	19.5%
1996–2000	50	23.3%
2001–2005	40	18.6%
2006–2010	38	17.7%
2011–2014	45	20.9%
Overall follow-up duration, months	75	(1–266)
Chief complaint leading to hospital admission		
Incidentaloma	21	9.8%
Anterior neck palpable mass	165	76.7%
Lateral neck palpable mass	7	3.3%
Neck discomfort	3	1.4%
Hoarseness	2	0.9%
Dyspnea	3	1.4%
Unknown	14	6.5%
Fine needle aspiration at admission		
Yes	161	74.9%
No	54	25.1%
Initial cytology		
Nondiagnostic or unsatisfactory	1	0.6%
Benign	23	14.3%
Atypia of undetermined significance or follicular lesion of undetermined significance	15	9.3%
Follicular neoplasm or suspicion of follicular neoplasm	17	10.6%
Suspicion of malignancy	11	6.8%
Malignant	92	57.1%
Others (schwannoma and abscess)	2	1.2%
Final pathology after thyroidectomy		
Benign	87	40.5%
Graves' disease (diffuse hyperplasia)	37	42.5%
Adenomatous goiter/nodular hyperplasia	26	29.9%
Hashimoto thyroiditis	2	2.3%
Follicular adenoma	18	20.7%
Hurthle cell adenoma	2	2.3%
Other (schwannoma and abscess)	2	2.3%
Malignant	128	59.5%
Papillary thyroid cancer	106	82.8%
Follicular thyroid cancer	21	16.4%
Papillary with follicular thyroid cancer	1	0.8%
Tumor size by pathology after thyroidectomy (cm)	2.5	(0.4–9.0)

**Table 2:** Clinicopathological features of the pediatric patients with thyroid nodules according to age group.

	0 to 10 (*n* = 24)		11 to 15 (*n* = 79)		16 to 18 (*n* = 112)		*p* value
Age, years (mean, SD)	8	±2.358	14	±1.300	17	±0.833	
Sex							0.002
Females	12	50.0%	61	77.2%	93	83.0%	
Males	12	50.0%	18	22.8%	19	17.0%	
Period of diagnosis							0.892
1990–1995	4	16.7%	17	21.5%	21	18.8%	
1996–2000	8	33.3%	16	20.3%	26	23.2%	
2001–2005	4	16.7%	12	15.2%	24	21.4%	
2006–2010	3	12.5%	16	20.3%	19	17.0%	
2011–2014	5	20.8%	18	22.8%	22	19.6%	
Chief complaint leading to hospital admission							0.197
Incidentaloma	3	12.5%	6	7.6%	12	10.7%	
Anterior neck palpable mass	18	75.0%	61	77.2%	86	76.8%	
Lateral neck palpable mass	2	8.3%	4	5.1%	1	0.9%	
Neck discomfort	0		2	2.5%	1	0.9%	
Hoarseness	0		2	2.5%	0		
Dyspnea	1	4.2%	1	1.3%	1	0.9%	
Unknown	0		3	3.8%	11	9.8%	
Fine needle aspiration at admission							0.008
Yes	20	83.3%	67	84.8%	74	66.1%	
No	4	16.7%	12	15.2%	38	33.9%	
Initial cytology							0.013
Nondiagnostic or unsatisfactory	1	5.0%	0		0		
Benign	5	25.0%	8	11.9%	10	13.5%	
Atypia of undetermined significance or follicular lesion of undetermined significance	2	10.0%	7	10.4%	6	8.1%	
Follicular neoplasm or suspicion of follicular neoplasm	2	10.0%	8	11.9%	7	9.5%	
Suspicion of malignancy	4	20.0%	5	7.5%	2	2.7%	
Malignant	6	30.0%	38	56.7%	48	64.9%	
Others (schwannoma and abscess)	0		1	1.5%	1	1.4%	
Final pathology after thyroidectomy							0.291
Benign	12	50.0%	27	34.2%	48	42.9%	
Graves' disease (diffuse hyperplasia)	0		7	25.9%	30	62.5%	
Adenomatous goiter/nodular hyperplasia	6	50.0%	11	40.7%	9	18.8%	
Hashimoto thyroiditis	0		0		2	4.2%	
Follicular adenoma	4	33.3%	7	25.9%	7	14.6%	
Hurthle cell adenoma	1	8.3%	1	3.7%	0		
Other (schwannoma and abscess)	1	8.3%	1	3.7%	0		
Malignant	12	50.0%	52	65.8%	64	57.1%	
Papillary thyroid cancer	9	75.0%	43	82.7%	54	84.4%	
Follicular thyroid cancer	3	25.0%	8	15.4%	10	15.6%	
Papillary with follicular thyroid cancer	0		1	1.9%	0		
Tumor size by pathology after thyroidectomy (cm)	2.6	(1.2–6.5)	2.5	(0.5–8.5)	2.6	(0.4–9.0)	0.587

**Table 3:** Clinicopathological features of the pediatric patients with thyroid nodules according to sex.

	Females (*n* = 166)		Males (*n* = 49)		*p* value
Age, years (mean, SD)	15	±2.748	13	±4.154	0.001
Age groups					0.002
0–10	12	7.2%	12	24.5%	
11–15	61	36.7%	18	36.7%	
16–18	93	56.0%	19	38.8%	
Period of diagnosis					0.149
1990–1995	33	19.9%	9	18.4%	
1996–2000	35	21.1%	15	30.6%	
2001–2005	35	21.1%	5	10.2%	
2006–2010	32	19.3%	6	12.2%	
2011–2014	31	18.7%	14	28.6%	
Chief complaint leading to hospital admission					0.037
Incidentaloma	13	7.8%	8	16.3%	
Anterior neck palpable mass	129	77.7%	36	73.5%	
Lateral neck palpable mass	3	1.8%	4	8.2%	
Neck discomfort	2	1.2%	1	2.0%	
Hoarseness	2	1.2%	0		
Dyspnea	3	1.8%	0		
Unknown	14	8.4%	0		
Fine needle aspiration at admission					0.032
Yes	119	71.7%	42	85.7%	
No	47	28.3%	7	14.3%	
Initial cytology					0.061
Nondiagnostic or unsatisfactory	0		1	2.4%	
Benign	15	12.6%	8	19.0%	
Atypia of undetermined significance or follicular lesion of undetermined significance	12	10.1%	3	7.1%	
Follicular neoplasm or suspicion of follicular neoplasm	11	9.2%	6	14.3%	
Suspicion of malignancy	7	5.9%	4	9.5%	
Malignant	73	61.3%	19	45.2%	
Others (schwannoma and abscess)	1	0.8%	1	2.4%	
Final pathology after thyroidectomy					0.410
Benign	66	39.8%	21	42.9%	
Graves' disease (diffuse hyperplasia)	34	51.5%	3	14.3%	
Adenomatous goiter/nodular hyperplasia	18	27.3%	8	38.1%	
Hashimoto thyroiditis	2	3.0%	0		
Follicular adenoma	11	16.7%	7	33.3%	
Hurthle cell adenoma	1	1.5%	1	4.8%	
Others (schwannoma and abscess)	0		2	9.5%	
Malignant	100	60.2%	28	57.1%	
Papillary thyroid cancer	83	83.0%	23	82.1%	
Follicular thyroid cancer	16	16.0%	5	17.9%	
Papillary with follicular thyroid cancer	1	1.0%	0		
Tumor size by pathology after thyroidectomy (cm)	2.5	(0.5–9.0)	2.6	(0.4–8.0)	0.353

**Table 4:** Clinicopathological features of the pediatric patients with thyroid nodules according to the period of diagnosis.

	1990 to 1995 (*n* = 42)		1996 to 2000 (*n* = 50)		2001 to 2005 (*n* = 40)		2006 to 2010 (*n* = 38)		2011 to 2014 (*n* = 45)		*p* value
Age, years (mean, SD)	15	±3.624	15	±3.138	15	±3.342	15	±2.857	15	±3.078	0.218
Age groups											0.892
0–10	4	9.5%	8	16.0%	4	10.0%	3	7.9%	5	11.1%	
11–15	17	40.5%	16	32.0%	12	30.0%	16	42.1%	18	40.0%	
16–18	21	50.0%	26	52.0%	24	60.0%	19	50.0%	22	48.9%	
Sex											0.149
Females	35	83.3%	35	70.0%	35	87.5%	32	84.2%	31	68.9%	
Males	9	21.4%	15	30.0%	5	12.5%	6	15.8%	14	31.1%	
Chief complaint leading to hospital admission								0.061			
Incidentaloma	0		1	2.0%	3	7.5%	6	15.8%	11	24.4%	
Anterior neck palpable mass	37	88.1%	42	84.0%	33	82.5%	26	68.4%	27	60.0%	
Lateral neck palpable mass	1	2.4%	2	4.0%	0		2	5.3%	2	4.4%	
Neck discomfort	0		0		0		2	5.3%	1	2.2%	
Hoarseness	0		1	2.0%	0		0		1	2.2%	
Dyspnea	1	2.4%	0		1	2.5%	0		1	2.2%	
Unknown	3	7.1%	4	8.0%	3	7.5%	2	5.3%	2	4.4%	
Fine needle aspiration at admission									≤0.00		
Yes	17	40.5%	36	72.0%	31	77.5%	36	94.7%	41	91.1%	
No	25	59.5%	14	28.0%	9	22.5%	2	5.3%	4	8.9%	
Initial cytology											≤0.00
Nondiagnostic or unsatisfactory	0		1	2.8%	0		0		0		
Benign	7	41.2%	6	16.7%	4	12.9%	3	8.3%	3	7.3%	
Atypia of undetermined significance or follicular lesion of undetermined significance	1	5.9%	1	2.8%	6	19.4%	2	5.6%	5	12.2%	
Follicular neoplasm or suspicion of follicular neoplasm	2	11.8%	8	22.2%	2	6.5%	2	5.6%	3	7.3%	
Suspicion of malignancy	2	11.8%	2	5.6%	1	3.2%	2	5.6%	4	9.8%	
Malignant	5	29.4%	16	44.4%	18	58.1%	27	75.0%	26	63.4%	
Others (schwannoma and abscess)	0		2	5.6%	0		0		0		
Final pathology after thyroidectomy											≤0.00
Benign	32	76.2%	24	48.0%	16	40.0%	5	13.2%	10	22.2%	
Graves' disease (diffuse hyperplasia)	13	40.6%	12	50.0%	7	43.8%	1	20.0%	4	40.0%	
Adenomatous goiter/nodular hyperplasia	13	40.6%	5	20.8%	3	18.8%	3	60.0%	2	20.0%	
Hashimoto thyroiditis	1	3.1%	1	4.2%	0		0		0		
Follicular adenoma	5	15.6%	2	8.3%	6	37.5%	1	20.0%	4	40.0%	
Hurthle cell adenoma	0		2	8.3%	0		0		0		
Other (schwannoma and abscess)	0		2	8.3%	0		0		0		
Malignant	10	23.8%	27	54.0%	24	60.0%	33	86.8%	35	77.8%	
Papillary thyroid cancer	8	80.0%	20	74.1%	20	83.3%	28	84.8%	29	82.9%	
Follicular thyroid cancer	2	20.0%	4	14.8%	4	16.7%	5	15.2%	6	17.1%	
Papillary and follicular thyroid cancer	0		1	3.7%	0		0		0		
Tumor size by pathology after thyroidectomy (cm)	2.5	(1.2–4.0)	2.5	(1.0–6.5)	3.0	(1.0–6.0)	2.5	(0.5–6.0)	2.6	(0.4–8.0)	0.849

## Data Availability

The retrospective research data used to support the findings of this study are available from the corresponding author upon request.
